# Identification of tumor antigens and immunogenic cell death-related subtypes for the improvement of immunotherapy of breast cancer

**DOI:** 10.3389/fcell.2022.962389

**Published:** 2022-10-25

**Authors:** Xi Cao, Xingtong Zhou, Chang Chen, Zhe Wang, Qiang Sun

**Affiliations:** Department of Breast Surgery, Peking Union Medical College Hospital, Peking Union Medical College, Chinese Academy of Medical Sciences, Beijing, China

**Keywords:** breast cancer, tumor antigens, immunogenic cell death, immunotherapy, prognosis

## Abstract

The current immunotherapy strategy for breast cancer is limited. Tumor neoantigens have been proven to be a promising biomarker and potential target of immunotherapy in a variety of tumors. However, their effectiveness for breast cancer remains unclear. Immunogenic cell death (ICD) is a regulated form of cell death that can reshape the tumor immune microenvironment and activate adaptive immune responses. To this end, we screened potential antigens that could be used both for the development of immunotherapy and differentiating the patient-specific immune responses based on ICD-related risk signatures, in order to formulate an accurate scheme for breast cancer immunotherapy. We retrieved the gene expression profiles of the breast invasive cancer cohort and their corresponding clinical control data from The Cancer Genome Atlas. The Gene Expression Profiling Interactive Analysis (GEPIA) database was used to evaluate tumor antigen expression, the cBioPortal program was used to identify genetic variations, and the TIMER website was used to estimate the immune infiltration signatures. The risk score predictive model based on the ICD-related genes was constructed using the least absolute shrinkage and selection operator (LASSO) Cox regression algorithm, and the cohort was divided into low- and high-risk score groups. Two tumor antigens, namely, *CCNE1* and *PLK1*, were associated with poor prognosis and infiltration of antigen-presenting cells. Furthermore, the ICD-related risk signature could significantly predict survival outcomes. The risk groups based on the ICD-related signature predictive model showed diverse immune infiltration and molecular and clinical features. The high-risk group was associated with low immune cell infiltration, immune score, expression of immune checkpoints, and human leukocyte antigen genes but high levels of *CCNE1* and *PLK1* and poor survival outcome. In conclusion, *CCNE1* and *PLK1* were identified as potential antigens in breast cancer. The ICD-related prognostic model distinguished immune response heterogeneity and predicted prognosis. Patients with high ICD-related risk scores were suitable to receive combination treatments based on *CCNE1* or *PLK1* and immune checkpoint inhibitors. In the future, these results will help us develop more accurate treatment schemes for patients with breast cancer.

## 1 Introduction

Breast cancer (BC) is a serious threat to women’s health worldwide, with the highest incidence and high death rate according to global cancer statistics 2020 (Sung et al., 2021). The global incidence rate and mortality of BC are also consistent with the epidemiological trends in Asian countries (Sung et al., 2021). Comprehensive treatment includes surgery, chemotherapy, radiotherapy, endocrine therapy, and targeted therapy. In recent years, immunotherapeutic drugs, such as programmed cell death protein 1 (PD-1) or programmed cell death ligand 1 (PD-L1) inhibitors combined with chemotherapy, have made a breakthrough in BC treatment. In 2019 and 2020, based on the results of IMpassion130 (Schmid et al., 2020a) and KEYNOTE-355 (Cortes et al., 2020) clinical trials, the FDA accelerated the approval of atezolizumab (PD-L1 inhibitor) and pembrolizumab (PD-1 inhibitor) combined with chemotherapy for locally advanced and metastatic triple-negative breast cancer (TNBC), respectively. After the KEYNOTE-522 trial in 2021 (Schmid et al., 2020b; Schmid et al., 2022), the FDA approved pembrolizumab in combination with chemotherapy as a new adjuvant and postoperative intensive treatment for high-risk early TNBC. However, these three combinatorial immunotherapies have not dramatically improved prognosis for patients with BC, potentially due to different treatment responses between patients and treatment tolerance. Therefore, there is an urgent need to develop an accurate immunotherapy strategy for patients with BC.

Immunogenic cell death (ICD) is a regulated form of cell death induced by stress that can activate cytotoxic T lymphocyte (CTL)-driven adaptive immunity and produce adaptive memory (Galluzzi et al., 2017). Viruses, bacteria, chemotherapy, radiotherapy, epigenetic modifiers, and target agents can all be cellular stressors associated with ICD (Galluzzi et al., 2020). ICD has three critical influencing factors: antigenicity, adjuvanticity, and microenvironmental factors (Galluzzi et al., 2020). Stress-related chemotherapy drugs include anthracyclines, DNA-damaging agents, poly (ADP-ribose) polymerase (PARP) inhibitors, and antimitotic agents, which are frequently used in the treatment of BC (Tesniere et al., 2010; Fucikova et al., 2011). Based on this information, PD-1 or PD-L1 inhibitors and traditional chemotherapy can be a powerful combination treatment for BC. However, researchers have not obtained satisfactory prognostic improvements for patients with BC using these treatments in clinical trials. Therefore, we urgently need to explore and analyze the ICD-related characteristics of breast cancer, further classify and describe population characteristics, and develop accurate immunotherapy strategies.

Malignant tumor cells show high antigenicity, largely due to the increased mutation rate that accompanies malignant cells in immune escape (Greaves, 2015; McGranahan and Swanton, 2017). Nonsynonymous mutations, genomic changes, and tumor neoantigens are retained and in turn activate *de novo* immune responses (Schumacher et al., 2019). Tumor neoantigens are important factors that affect the antigenicity of ICD, and they evolve spatially and temporally, resulting in antigen heterogeneity (Greaves, 2015). The antigenicity of tumor cells is also affected by post-translational modifications regulated by the tumor microenvironment (TME) (Malaker et al., 2017). Tumor antigens could be used as both a biomarker and target for tumor immunotherapy such as the tumor vaccine that does not need to enter the nucleus to change the genome (Grunwitz and Kranz, 2017). In recent years, research studies on the application of tumor vaccines in gastrointestinal cancer (Cafri et al., 2020), prostate cancer (Rausch et al., 2014), and melanoma (Wang et al., 2018) have achieved satisfactory results. A recent study showed that EV-ligand-dependent corepressor mRNA therapy in combination with PD-L1 inhibitor overcame resistance and metastasis in preclinical breast cancer models (Pérez-Núñez et al., 2022).

Therefore, the purpose of this study was to identify novel breast cancer antigens that could be used as biomarker and targets and to classify breast cancer patients according to ICD-associated characteristics to identify breast cancer patients who could benefit from a combination of immunotherapy and chemotherapy.

## 2 Materials and methods

### 2.1 Patients and datasets

The RNA sequencing (RNA-seq) gene expression data of breast invasive cancer cohort (BRCA) tissue samples (*n* = 1101) with clinical features and matched adjacent normal tissue samples (*n* = 113) were downloaded from The Cancer Genome Atlas (TCGA) dataset^1^. RNA-seq data were normalized and integrated using R software v4.0.3. The overall survival (OS) and disease-free survival (DFS) of the BRCA cohort were assessed using the Kaplan–Meier method with the log-rank test using the survival package.

### 2.2 Gene Expression Profiling Interactive Analysis database analysis

The website Gene Expression Profiling Interactive Analysis (GEPIA2)^2^ (Tang et al., 2017) was used for gene expression analysis. Differentially expressed genes (DEGs) between breast cancer and normal tissues were explored by ANOVA with |Log2FC|>1 and q < 0.01 and plotted on the chromosome.

### 2.3 cBioPortal database analysis

The cBioPortal^3^ (Cerami et al., 2012) was used to explore and visualize the genetic variation, including copy number variation (CNV) and mutation in the breast invasive cancer cohort (TCGA, PanCancer Atlas, *n* = 1084). Both amplified and mutated genes with frequencies of > 0.1% were selected.

### 2.4 TIMER database analysis

The TIMER website^4^ (Li et al., 2017) was used to estimate and visualize the correlation between screened breast cancer antigens and infiltration abundance of six immune cells, namely, B cells, CD4^+^ T cells, CD8^+^ T cells, neutrophils, macrophages, and dendritic cells (DCs).

### 2.5 Construction of immunogenic cell death-related prognostic model

ICD-related genes were obtained by searching and summarizing extensive literature (Garg et al., 2015). The least absolute shrinkage and selection operator (LASSO) Cox regression algorithm was used to construct a prognostic model based on ICD-related genes with prognostic significance, using the R software package glmnet (Bøvelstad et al., 2007). The formula for calculating the ICD-related risk score is as follows:

Risk score=(0.0162)×NT5E+(0.0675)×ATG5+(0.0221)×PIK3CA+(0.0352)×IL1R1+(−0.0118)×IL1B+(0.2049)×HSP90AA1+(0.0342)×EIF2AK3+(−0.2703)×MYD88+(0.5396)×IL10+(−0.0987)×CD8A+(−0.1254)×IFNG,

Risk score = sum of coefficients × gene expression level. The coefficients were calculated using the LASSO-Cox model.

### 2.6 Tumor microenvironment immune component analysis

The signatures of immune components, including the types and abundance of immune cells and immune and stromal scores, were assessed using xCell algorithms in the R immunedeconv package. Immune checkpoint-related gene expression levels were also determined.

### 2.7 Tumor Immune Dysfunction and Exclusion database analysis

Tumor Immune Dysfunction and Exclusion (TIDE)^5^ analysis, based on the mechanism that T-cell dysfunction and T-cell infiltration inhibited in tumors with the low CTL level, was performed to estimate the immunotherapy response in the BRCA cohort.

### 2.8 Statistical analysis

The Wilcoxon rank sum test and Kruskal–Wallis one-way analysis of variance were used to compare data between the two groups and three groups, respectively. The Kaplan–Meier method with log-rank test and univariate Cox regression analysis was used for survival analysis. A two-sided *p* < 0.05 was considered statistically significant.

## 3 Results

### 3.1 Exploring potential tumor antigens of breast cancer

**FIGURE 1:**
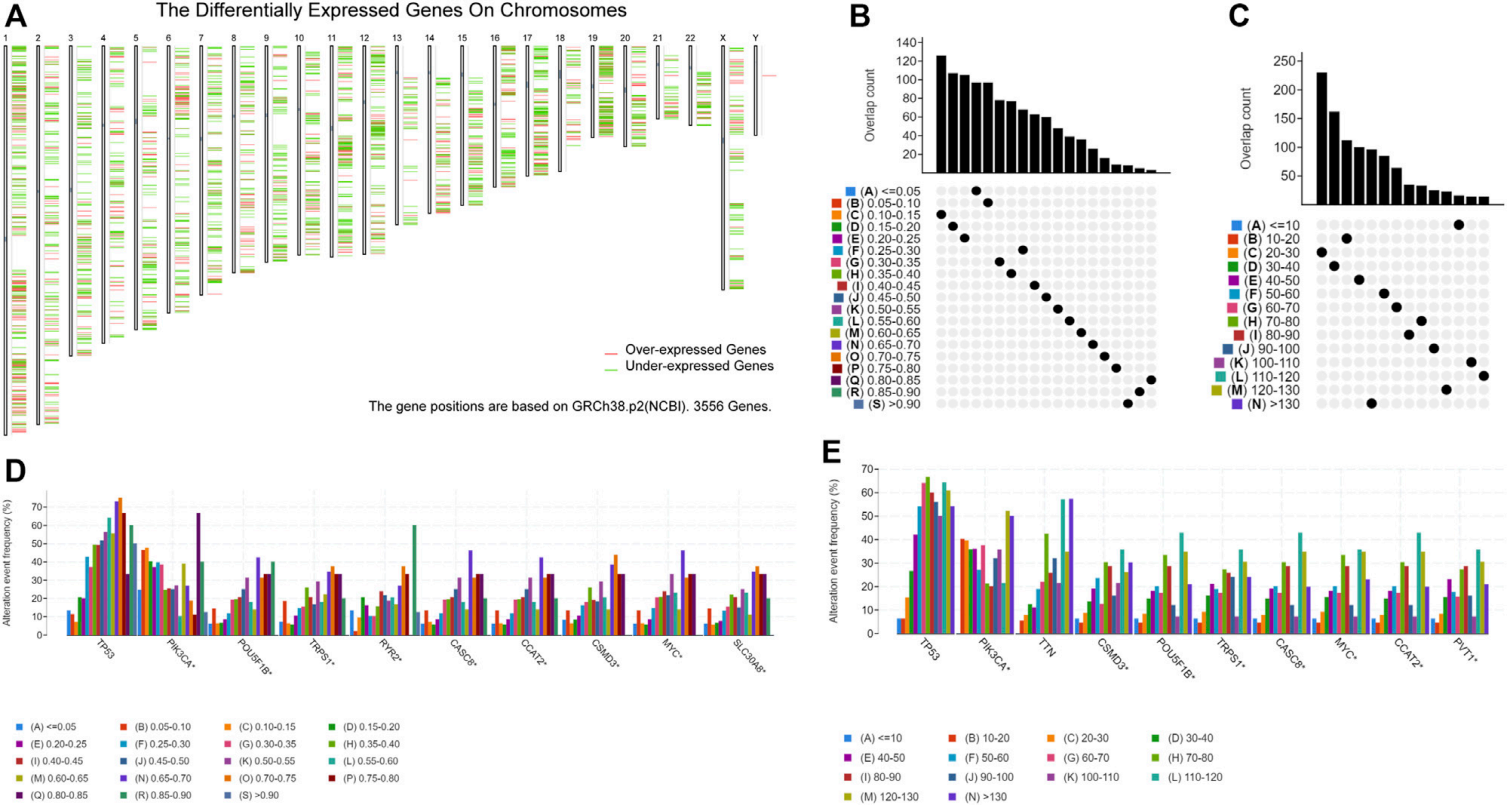
Identification of potential tumor-associated antigens of breast cancer (BC). **(A)** Chromosomal distribution of upregulated and downregulated genes in BC. **(B)** Overlapping samples in the altered genome fraction group. **(C)** Overlapping samples in the mutation count group. **(D)** Highest average frequency genes in the fraction genome altered genome. **(E)** Highest average frequency genes in the mutation count group.

GEPIA2 analysis revealed that 1418 genes among 3556 DEGs were overexpressed in breast cancer tissue compared with normal tissue, which could potentially encode tumor neoantigens (Figure 1A) (Supplementary Table S1). Based on the results of cBioPortal analysis (Supplementary Table S1), 20,099 amplified genes with frequencies > 0.1% in breast cancer were identified. In the fraction genome altered group, the genes with the highest alteration frequency included *TP53*, *PIK3CA*, *POU5F1B*, *TRPS1*, *RYR2*, *CASC8*, *CCAT2*, *CSMD3*, *MYC*, and *SLC30A8* (Figures 1B,D). According to the results, 13,407 mutated genes with frequencies > 0.1% of breast cancer were identified (Supplementary Table S1). In the mutation count group, 10 genes with the highest alteration event frequency, *TP53*, *PIK3CA*, *TTN*, *CSMD3*, *POU5F1B*, *TRPS1*, *CASC8*, *MYC*, *CCAT2*, and *PVT1*, were detected (Figures 1C,E). We found that *TP53*, *PIK3CA*, *CSMD3*, *POU5F1B*, *TRPS1*, *CASC8*, *MYC*, and *CCAT2* were the most frequently altered genes in both the fraction genome alteration and tumor mutational count groups (Figures 1D,E). Collectively, 701 overexpressed, amplified, and mutated genes were identified for further analyses.

### 3.2 Correlation of tumor antigens with breast cancer prognosis and immune infiltration

**FIGURE 2:**
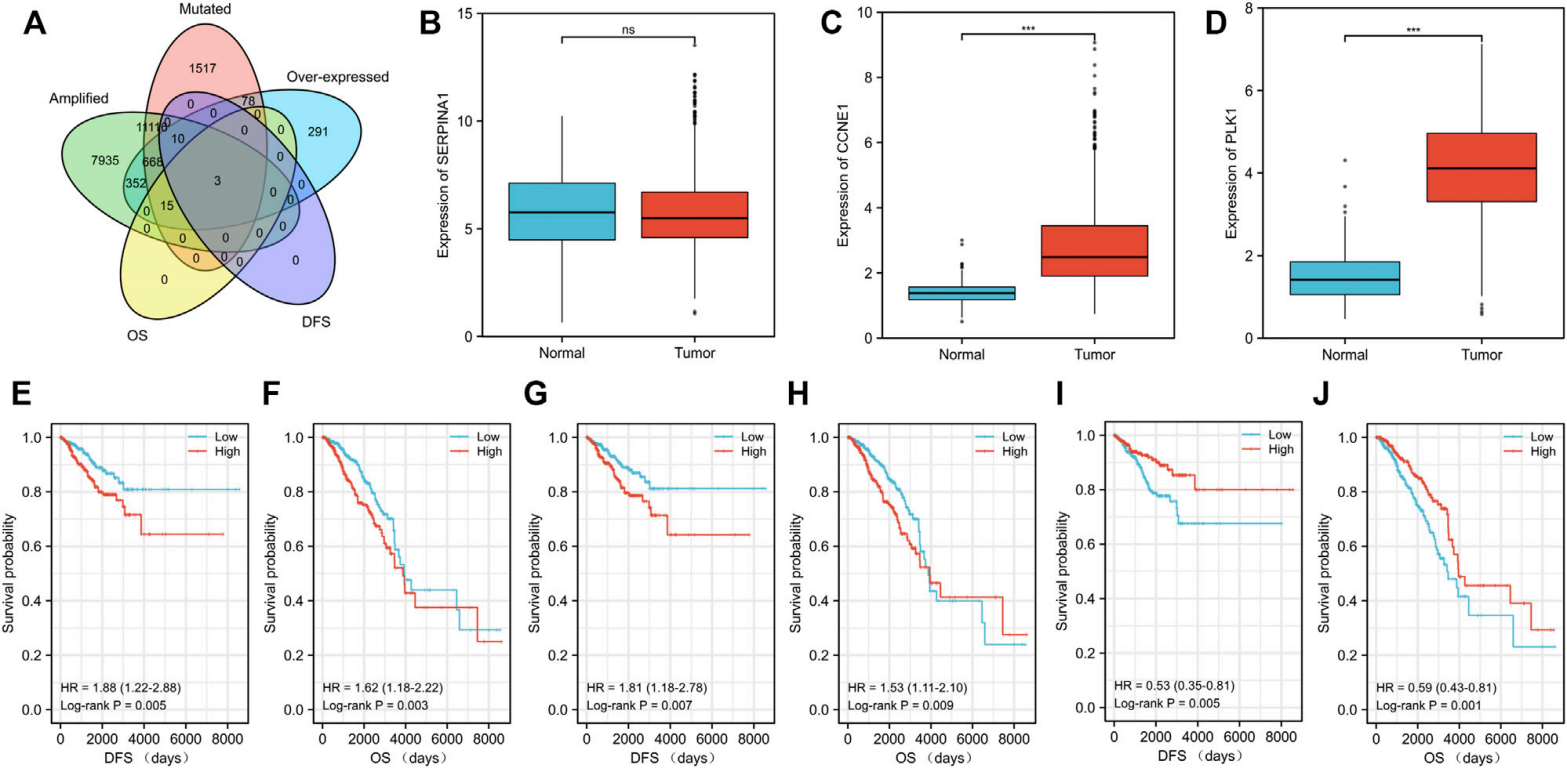
Identification of tumor antigens related to the prognosis of patients with breast cancer (BC). **(A)** Venn diagram showing the potential tumor antigens (total 701) with mutation, amplification and overexpression, and significant association with both disease-free survival (DFS) and overall survival (OS) (*p* < 0.01) (total three hub genes). Differential expression levels of three hub genes including **(B)** *SERPINA1*, **(C)** *CCNE1*, and **(D)** *PLK1* between breast cancer tissue and normal tissue. The prognostic value of three potential antigens. The Kaplan–Meier curves showing the DFS of patients with breast cancer stratified based on the expression level of **(E)** *CCNE1*, **(G)** *PLK1,* and **(I)** *SERPINA1* and the OS on the basis of **(F)** *CCNE1*, **(H)** *PLK1*, and **(J)** *SERPINA1*. The cohort was divided into low- and high-risk groups with median expression as the cutoff. P< 0.05 was considered statistically significant.

**FIGURE 3:**
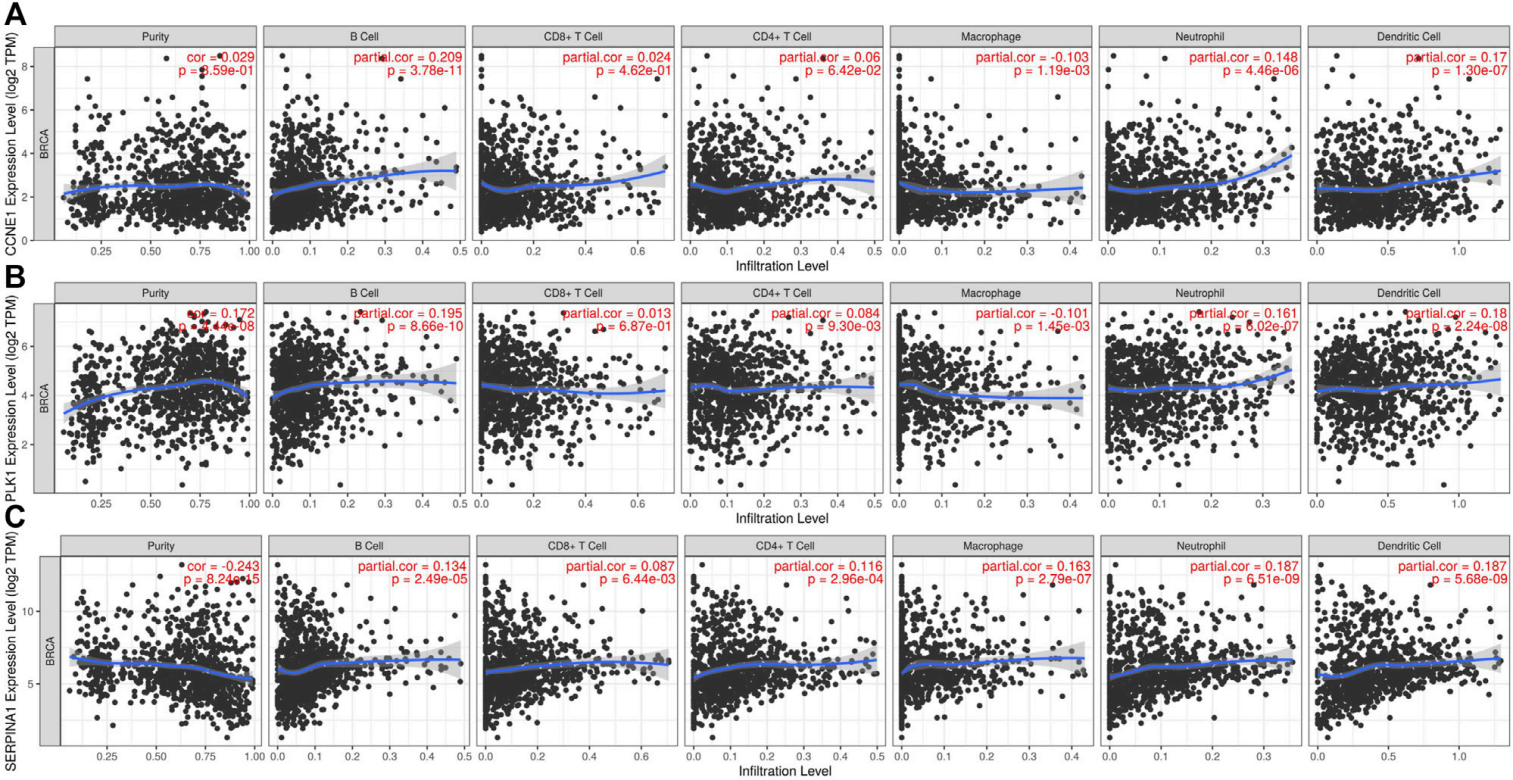
Identification of tumor antigens associated with immune cell infiltration. Correlation between the expression levels of **(A)** *CCNE1*, **(B)** *PLK1*, and **(C)** *SERPINA1* and infiltration level of immune cells (B cells, CD8^+^ T-cell, CD4^+^ T-cells, macrophages, neutrophils, and dendritic cells).

Tumor antigens associated with DFS (*p* < 0.01) (Supplementary Table S2) and OS (*p* < 0.01) (Supplementary Table S2) were selected from the 701 genes. Three hub genes, namely, cyclin E1 (*CCNE1*), polo-like kinase 1 (*PLK1*), and serpin family A member 1 (*SERPINA1*), were strongly associated with both DFS (*p* < 0.01) and OS (*p* < 0.01) in BC (Figure 2A). Both *CCNE1* (*p* < 0.001) (Figure 2C) and *PLK1* (*p* < 0.001) (Figure 2D) showed higher expression in tumor tissue than in normal tissue, while the expression of *SERPINA1* in both tumor and normal tissue was not significantly different (*p* = 0.541) (Figure 2B) (Supplementary Table S3). High *CCNE1* expression was associated with worse DFS (hazard ratio [HR] = 1.88, 95% confidence interval [CI]: 1.22–2.88, log-rank test, *p* = 0.005) (Figure 2E) and OS (HR = 1.62, 95% CI: 1.18–2.22, log-rank test, *p* = 0.003) (Figure 2F) in BC. High *PLK1* expression was correlated with both worse DFS (HR = 1.81, 95% CI: 1.18–2.78, log-rank test, *p* = 0.007) (Figure 2G) and OS (HR = 1.53, 95% CI:1.11–2.10, log-rank test, *p* = 0.009) (Figure 2H). In contrast, high *SERPINA1* expression was related to both longer DFS (HR = 0.53, 95% CI: 0.35–0.81, log-rank test, *p* = 0.005) (Figure 2I) and OS (HR = 0.59, 95% CI: 0.43–0.81, log-rank test, *p* = 0.001) (Figure 2J). We also estimated the correlation between the immune cell infiltration and the three hub genes, namely, *CCNE1*, *PLK1*, and *SERPINA1*. Based on the TIMER algorithm, expression levels of both *CCNE1* and *PLK1* were positively related to the abundance of B cells (*CCNE1*: r = 0.209, *p* < 0.001; *PLK1*: r = 0.195, *p* < 0.001) and DCs (*CCNE1*: r = 0.17, *p* < 0.001; PLK1: r = 0.18, *p* < 0.001) (Figures 3A,B), while the expression level of *SERPINA1* was positively correlated with B cells (r = 0.134, *p* < 0.001), macrophages (r = 0.163, *p* < 0.001), and DCs (r = 0.187, *p* < 0.001) (Figure 3C). Additionally, we found that these hub genes were positively related to neutrophil abundance (*CCNE1*: r = 0.148, *p* < 0.001; *PLK1*: r = 0.161, *p* < 0.001; *SERPINA1*: r = 0.187, *p* < 0.001).

### 3.3 Characteristics of immunogenic cell death-related genes in a breast cancer cohort

**FIGURE 4:**
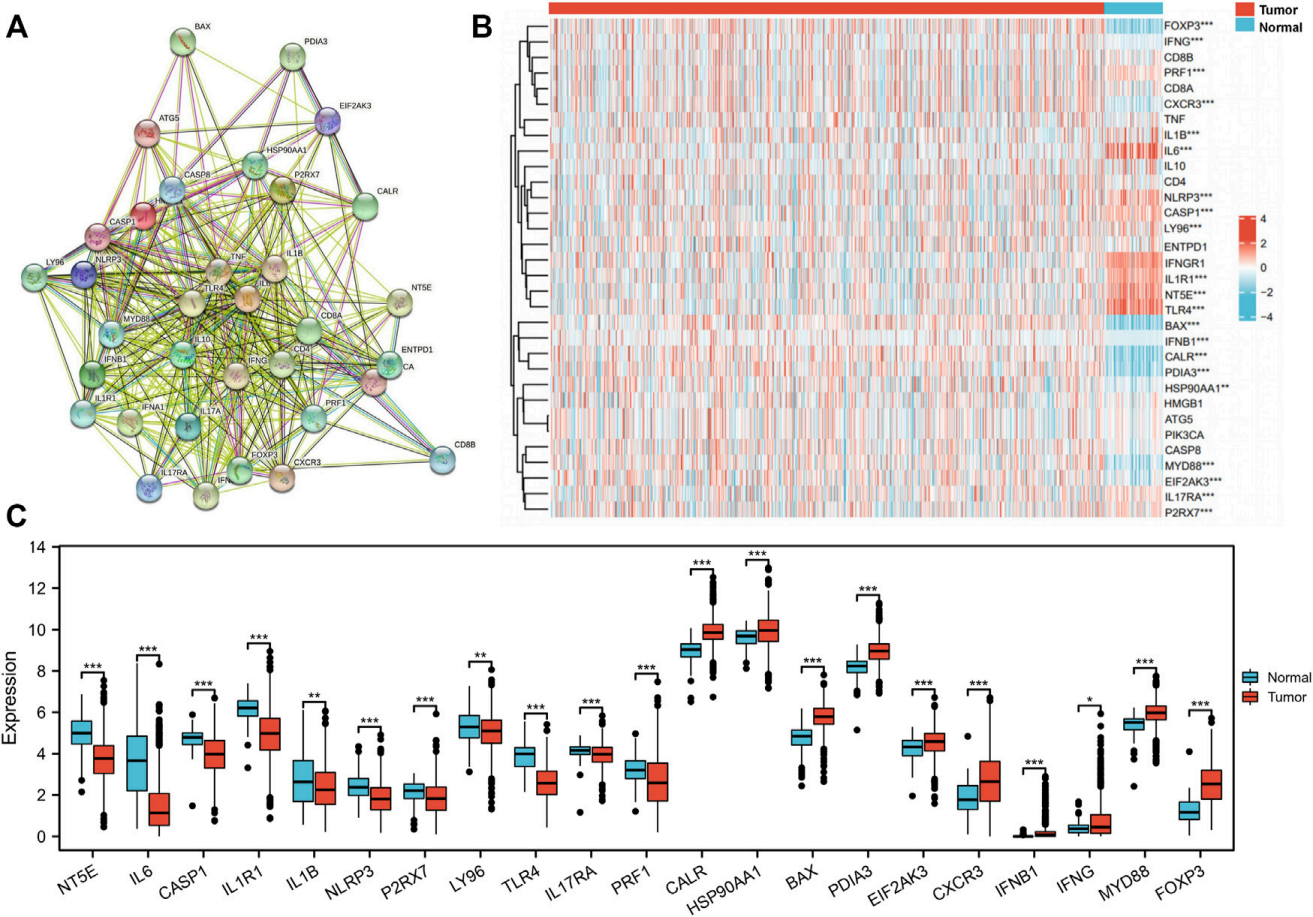
Identification of immunogenic cell death (ICD)-related gene expression pattern in The Cancer Genome Atlas (TCGA) breast invasive cancer (BRCA) cohort. **(A)** Protein–protein interactions among the 34 ICD-associated genes (number of nodes: 34, number of edges: 332, average node degree: 19.5, average local clustering coefficient: 0.8, expected number of edges: 62, and PPI enrichment *p*-value: <1.0e-16). **(B)** Heatmap of 32 ICD-related gene expression between tumor and normal tissue in the TCGA BRCA cohort. **(C)** In total, 21 genes with different expression patterns between tumor and normal tissues in the TCGA BRCA cohort. *p < 0.05, **p < 0.01, ***p < 0.001.

The 34 ICD-related genes have been previously identified and summarized based on an extensive literature search (Garg et al., 2015). The connections between these ICD-related genes were explored by the protein–protein interaction (PPI) network analysis using the STRING database (Figure 4A) (Supplementary Table S4). The network analysis showed that the local clustering coefficient was 0.796 and the interactions among these genes were both significantly and biologically connected (*p* < 1.0e-16). Furthermore, we compared the expression patterns of ICD-related genes between tumor and normal tissues in the TCGA BRCA cohort. As *IL17A* and *IFNA1* were not expressed in most BC samples, they were not included in further analysis. The expression levels of 21 of the 32 genes were notably different between tumor and normal tissues (Figure 4B) (Supplementary Table S5). Eleven genes *NT5E*, *IL6*, *CASP1*, *IL1R1*, *IL1B*, *NLRP3*, *P2RX7*, *LY96*, *TLR4*, *IL17RA*, and *PRF1* were expressed at low levels in the tumor tissues, while 10 genes *CALR*, *HSP90AA1*, *BAX*, *PDIA3*, *EIF2AK3*, *CXCR3*, *IFNB1*, *IFNG*, *MYD88*, and *FOXP3* were overexpressed in the tumor tissues.

### 3.4 Construction of the immunogenic cell death-related prognostic model in the breast cancer cohort

**FIGURE 5:**
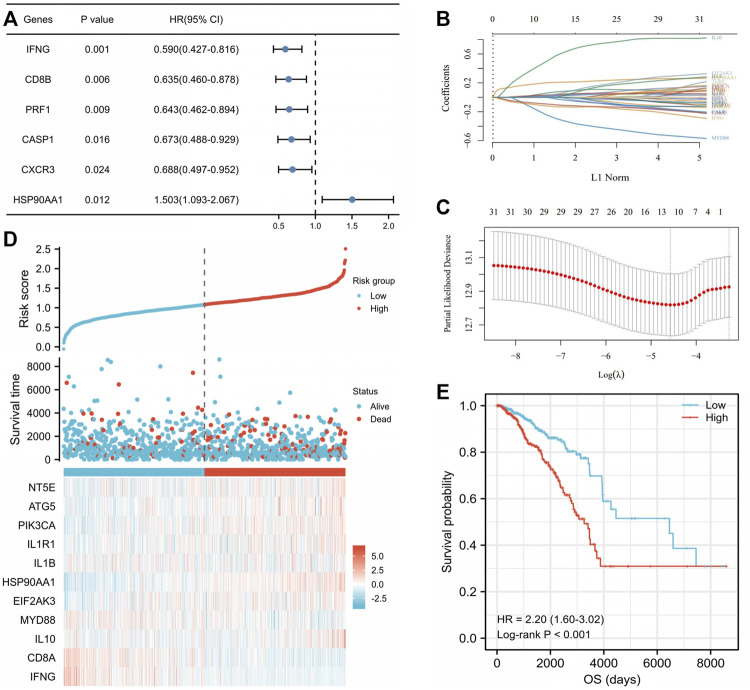
Construction of the immunogenic cell death (ICD)-related prognostic signatures in The Cancer Genome Atlas (TCGA) breast invasive cancer (BRCA) cohort. **(A)** Univariate Cox analysis examined the prognostic ability of the ICD-related genes in overall survival (OS) of the TCGA BRCA cohort. The coefficient of the 11 selected genes was displayed by the lambda parameter in which the abscissa represented the lambda value of independent variable and the ordinate represented the coefficient of the independent variable **(B)**. Eleven selected genes were with the lowest partial likelihood deviance plotted using the LASSO-Cox regression model **(C)**. **(D)** Distribution of risk score, survival time, and status and heatmap of 11 ICD-related prognostic genes in the cohort. The top scatterplot with different colors represent the risk score from low to high in different groups. The scatterplot distribution represents the risk score of different samples corresponding to the survival time and survival status. The heatmap showed the signature of the gene expression. **(E)** Kaplan–Meier curves by log-rank test and univariate Cox regression analysis showed that the low-risk score group had a significantly higher OS compared to the high-risk score group (HR = 2.20, 95% CI: 1.60–3.02, *p* < 0.001).

Correlations between 32 ICD-related genes and OS were analyzed by univariate Cox regression analysis with a significance threshold of *p* < 0.05 (Supplementary Table S6). High expression of *IFNG* (HR = 0.590, 95% CI: 0.427–0.816, *p* = 0.001), *CD8B* (HR = 0.635, 95% CI: 0.460–0.878, *p* = 0.006), *PRF1* (HR = 0.643, 95% CI: 0.462–0.894, *p* = 0.009), *CASP1* (HR = 0.673, 95% CI: 0.488–0.929, *p* = 0.016), and *CXCR3* (HR = 0.688, 95% CI: 0.497–0.952, *p* = 0.024) was associated with longer OS, while *HSP90AA1* (HR = 1.503, 95% CI: 1.093–2.067, *p* = 0.012) was associated with lower OS (Figure 5A). We then constructed a prognostic model based on the expression values of the 32 ICD-related genes using LASSO regression analysis. Eleven hub genes were identified with the lowest partial likelihood deviance (PLD) (Figures 5B,C). The patients were divided into high- and low-risk groups based on the median score (Supplementary Table S7). We then explored the relationship between the risk score and OS time. We found a significantly higher survival rate in the low-risk group than that in the high-risk group (Figure 5D), and the OS of the high-risk group was significantly shorter than that of the low-risk group (HR = 2.20, 95% CI: 1.60–3.02, log-rank test, *p* < 0.001) (Figure 5E).

### 3.5 Correlation of immunogenic cell death risk signature with breast cancer tumor microenvironments

**FIGURE 6:**
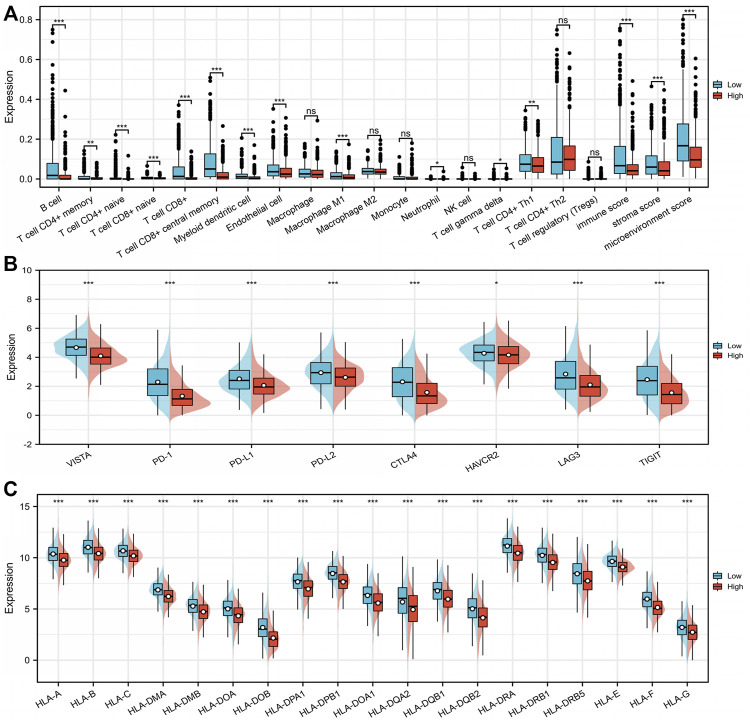
Characteristics of the immune microenvironment between low- and high-immunogenic cell death (ICD)-related risk groups in The Cancer Genome Atlas (TCGA) breast invasive cancer (BRCA) cohort. The expression difference of **(A)** immune cell infiltration levels, **(B)** immune checkpoint genes, and **(C)** human leukocyte antigen (HLA) genes between ICD-related risk groups. *p < 0.05, **p < 0.01, and ***p < 0.001.

**FIGURE 7:**
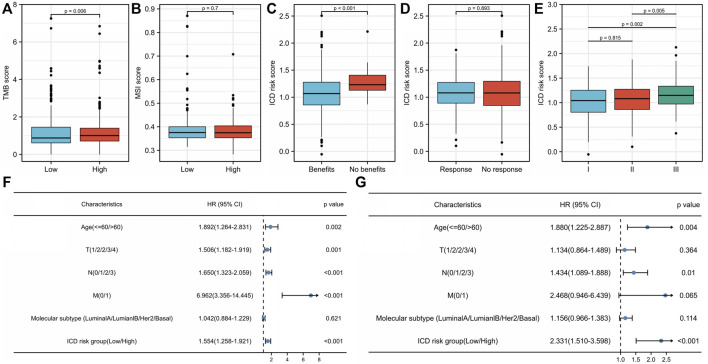
Association of the immunogenic cell death (ICD) risk signature with clinical characteristics in The Cancer Genome Atlas (TCGA) breast invasive cancer (BRCA) cohort. The **(A)** tumor mutational burden (TMB) score and **(B)** microsatellite instability (MSI) score in low- and high-ICD-related risk groups. The ICD risk score between **(C)** benefit and no benefit groups, **(D)** response and no response groups based on Tumor Immune Dysfunction and Exclusion (TIDE) analysis. **(E)** Difference of ICD risk score in different cancer stages. **(F)** Univariate and **(G)** multivariate Cox analyses evaluate the independent prognostic value of the ICD risk signature in patients with breast cancer.

There were significant differences in TME between the low- and high-risk score groups. The low-risk group had much higher infiltrating levels of B cells, T-cell CD4^+^ naive, T-cell CD8^+^ naive, T-cell CD8^+^, T-cell CD8^+^ central memory, myeloid dendritic cells, endothelial cells, macrophage M1 (*p* < 0.001), T-cell CD4^+^ memory and T-cell CD4^+^ Th1 (*p* < 0.01), and neutrophils and γδ T cells (*p* < 0.05) than the high-risk group (Figure 6A) (Supplementary Table S8). The low-risk group had significantly higher immune, stromal, and microenvironment scores (*p* < 0.001) than those of the high-risk group (Figure 6A). The expression levels of *VISTA*, *PD-1*, *PD-L1*, *PD-L2*, *CTLA4*, *LAG3*, and *TIGIT* in the low-risk group were significantly higher than those in the high-risk group (*p* < 0.0001) (Figure 6B) (Supplementary Table S8). In addition, most human leukocyte antigen (HLA) genes (*p* < 0.0001) (Figure 6C) (Supplementary Table S8) were upregulated in the low-risk group. The tumor mutational burden (TMB) score in the low-risk group was lower than that in the high-risk group (*p* = 0.006) (Figure 7A) (Supplementary Table S9), whereas the microsatellite instability (MSI) score was not significantly different between the low- and high-risk groups (*p* = 0.7) (Figure 7B) (Supplementary Table S9). Further analysis based on TIDE to estimate the predictive effect of the ICD risk signature on the potential efficacy of immunotherapy showed that the ICD risk score was significantly higher in the no benefit group than that in the benefit group (*p* < 0.001) (Figure 7C), while there was no difference between the response and no response groups (*p* = 0.893) (Figure 7D) (Supplementary Table S9). These results indicate that patients with low ICD risk scores may benefit from immunotherapy.

### 3.6 Association of immunogenic cell death risk signature with breast cancer clinical characteristics

We further compared ICD risk scores among different clinical stages in the TCGA BRCA cohort. The ICD risk score was significantly higher in stage III than that in both stage I (*p* = 0.002) and stage II (*p* = 0.005), whereas there was no difference between stages I and II (*p* = 0.815) (Figure 7E) (Supplementary Table S10). Univariate and multivariate Cox analyses were performed to estimate the prognostic value of the ICD risk signature. The univariate analysis indicated that the high-risk score group was significantly associated with shorter OS (HR = 1.554, 95% CI: 1.258–1.921, *p* < 0.001) (Figure 7F) (Supplementary Table S10). Multivariate analysis showed that the ICD risk score could serve as an independent prognostic factor for patients with BC (HR = 2.331, 95% CI: 1.510–3.598, *p* < 0.001) (Figure 7G).

### 3.7 Relationship between tumor antigens and immunogenic cell death risk signature in breast cancer

**FIGURE 8:**
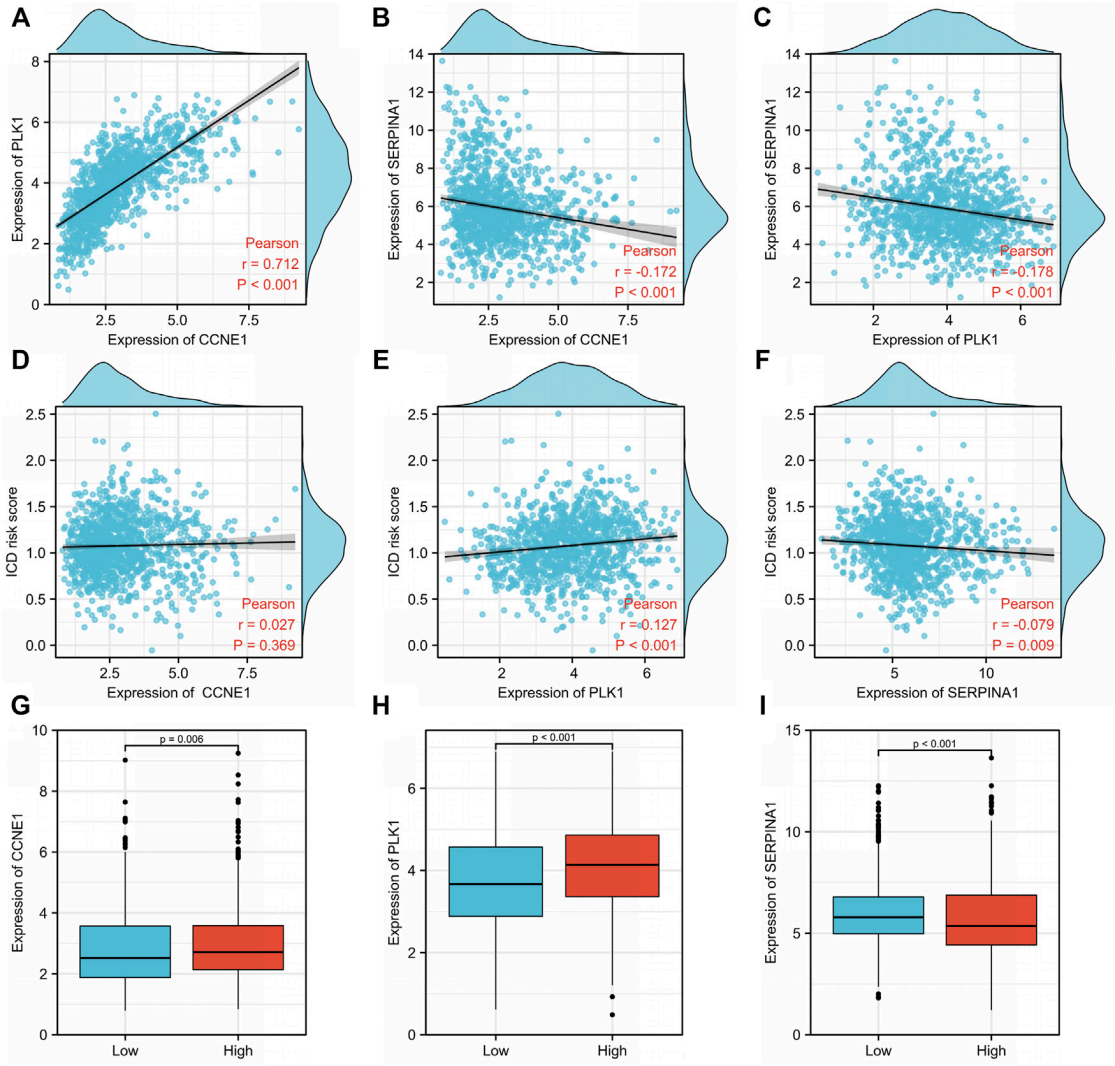
Relationship between tumor antigens and immunogenic cell death (ICD) risk signature. Pearson analyses between expression of **(A)** *CCNE1* and *PLK1*, **(B)** *CCNE1* and *SERPINA1*, and **(C)** *PLK1* and *SERPINA1*. Correlation between the ICD risk score and **(D)** *CCNE1*, **(E)** *PLK1*, and **(F)** *SERPINA1*. Expression levels of **(G)** *CCNE1*, **(H)** *PLK1*, and **(I)** *SERPINA1* in low- and high-ICD-related risk groups.

We then explored the relationship between tumor antigens (*CCNE1*, *PLK1*, and *SERPINA1*) and ICD risk score using Pearson correlation analysis. *CCNE1* expression was positively correlated to *PLK1* (r = 0.712, *p* < 0.001) (Figure 8A) but negatively correlated to *SERPINA1* (r = −0.172, *p* < 0.001) (Figure 8B). There was a negative correlation between *PLK1* and *SERPINA1* expressions (r = −0.178, *p* < 0.001) (Figure 8C). The ICD risk score was positively and weakly related to *PLK1* (r = 0.127, *p* < 0.001) (Figure 8E), while it was negatively and weakly related to *SERPINA1* (r = −0.079, *p* = 0.009) (Figure 8F). There was no significant correlation with *CCNE1* (r = 0.027, *p* = 0.369) (Figure 8D). *CCNE1* (*p* = 0.006) (Figure 8G) and *PLK1* expressions (*p* < 0.001) (Figure 8H) were significantly higher in the high-risk group, while *SERPINA1* (*p* < 0.001) (Figure 8I) was lower in the high-risk group compared to the low-risk group.

## 4 Discussion

Breast cancer is a threat to women’s health worldwide. In recent years, combining immunotherapies, such as PD-1 and PD-L1 inhibitors, with chemotherapy has been a breakthrough in TNBC treatment. However, limited immunotherapy tolerance and heterogenous immune compositions have led to limited therapeutic effects. In this study, we explored and analyzed potential tumor antigens in patients with breast cancer and classified them according to ICD-related risk characteristics. These antigens can be combined to identify immunotherapy targets and cluster patients that have a higher likelihood of positive response to immunotherapy.

Tumor neoantigens are externally expressed peptides encoded by non-synonymous polymorphic genes introduced by somatic mutations that accumulate during tumor progression (Yarchoan et al., 2017). Tumor neoantigens are recognized by new antigen-specific T-cell receptors (TCRs) of the major histocompatibility complexes (MHCs) and activate the *de novo* immune response (Schumacher et al., 2019). Future directions may involve investigating candidates for immunotherapy development through further analyses and screening.

In this study, we found that the hub genes *CCNE1*, *PLK1*, and *SERPINA1* were significantly overexpressed, amplified, and mutated in breast cancer tissues compared with normal tissues. Both *CCNE1* and *PLK1* were highly expressed and were related to worse survival outcomes in breast cancer, indicating that they are suitable candidate targets for cancer treatment.

*CCNE1* is a gene on 19q12 that is amplified in a variety of malignant tumors, associated with genomic instability and resistance to cytotoxic therapy (Zack et al., 2013; Yuan et al., 2018; Watkins et al., 2020). Previous studies have shown that *CCNE1* amplification is associated with poor survival in TNBC (Zhao et al., 2019; Yuan et al., 2021). miR-195-5p or miR-497-5p may promote TNBC progression by regulating *CCNE1* expression (Yang et al., 2019; Liu et al., 2021). The PALOMA-3 trial showed that in hormone-receptor-positive breast cancer, palbociclib efficacy was lower in the group with high levels of *CCNE1* mRNA, especially in patients with metastasis, compared with low *CCNE1* expression (Turner et al., 2019). However, *CCNE1* amplification has little relationship with human epidermal growth factor receptor 2 (HER2)-positive breast cancer prognosis and anti-HER2 targeted therapy efficacy (Luhtala et al., 2016). This may indicate that *CCNE1* may not be relevant in all subtypes of BC.

Polo-like kinases (PLKs) are a class of serine/threonine protein kinases containing five family members (Golsteyn et al., 1996). PLK1, a regulatory mitotic protein kinase, plays important roles in cell division and genome stability, and overexpression causes dysfunctional cell growth and oncogenesis (Shakeel et al., 2021). Previous research has also shown that patients with breast cancer having high *PLK1* expression had significantly worse survival outcomes, especially with TP53 mutations, than those with low *PLK1* expression (King et al., 2012). Further research also showed that high *PLK1* expression was related to shorter metastasis-free survival and poor response to endocrine therapy in hormone-receptor-positive breast cancer, whereas PLK1 inhibition resulted in tumor shrinkage and acquired palbociclib resistance (Montaudon et al., 2020). PLK1 inhibition also sensitizes breast cancer cells to radiation by suppressing autophagy (Wang et al., 2021).

Further immune infiltration analyses showed that *CCNE1* and *PLK1* were both positively correlated with the abundance of APCs, such as B cells and DCs. Based on our results and the characteristics of *CCNE1* and *PLK1*, they can be appropriate candidates for mRNA vaccines. Although *SERPINA1* was overexpressed, amplified, and mutated, the difference in its expression between breast cancer and normal tissues was not significant.

ICD is a type of regulated cell death that activates an antigen-specific adaptive immune response. Chemotherapy and radiotherapy are stressors that lead to the release and relocation of damage-associated molecular patterns (DAMPs), promote the recruitment of APCs, induce phagocytosis by DCs, and produce inflammatory chemokines, thereby activating ICD and enhancing the antitumor immune response (Fucikova et al., 2020). The most important ICD-related DAMPs are surface-exposed calreticulin (CALR), secreted ATP (Di Virgilio et al., 2018; Fucikova et al., 2021), annexin A1 (ANXA1) (Baracco et al., 2019), type I interferon, and high mobility group box 1 (HMGB1) (Zhu et al., 2021). We could evaluate the basic ICD level of patients diagnosed with breast cancer by detecting the mRNA and protein levels of the aforementioned markers and provide a basis for further immunotherapy strategies. Recently, metal-based complexes that use Pt, Ru, Ir, Cu, and Au have been studied as ICD inducers (Sen et al., 2022). Research based on a large-scale meta-analysis summarized 34 key genes associated with ICD as follows: *ENTPD1*, *NT5E*, *HMGB1*, *ATG5*, *PIK3CA*, *IFNA1*, *IL6*, *CASP1*, *IL1R1*, *IL1B*, *NLRP3*, *P2RX7*, *LY96*, *TLR4*, *IFNGR1*, *IL17RA*, *PRF1*, *CALR*, *HSP90AA1*, *BAX*, *PDIA3*, *EIF2AK3*, *CXCR3*, *IFNB1*, *MYD88*, *FOXP3*, *IL10*, *CASP8*, *TNF*, *CD4*, *CD8A*, *CD8B*, *IFNG*, and *IL17A* (Garg et al., 2015). Also, we used these 34 key genes in this research.

In this study, we constructed a prognostic risk signature using 11 ICD-related genes and stratified the breast cancer cohort into low- and high-risk score groups. A high-ICD-related risk score was significantly associated with worse DFS and OS and could be an independent predictive index for patients with breast cancer. The high-risk score group was also related to low levels of immune infiltration, low immune, stromal, and TME scores and related to low immune checkpoint and HLA gene expression, all of which are consistent with a tumor that is unlikely to trigger an immune response. In contrast, the low-risk group had a higher TME score, abundant immune cell infiltration, high immune checkpoint and HLA gene expression and are more likely to benefit from immunotherapy, all of which are consistent with tumors that can trigger immune responses. Based on the high immune score in the low-ICD-risk score group, chemotherapy or radiotherapy alone can induce a strong ICD to activate adaptive anti-tumor immunity. Combining immunotherapy, such as PD-1/PD-L1 inhibitors, and cytotoxic therapy can achieve a stronger effect. Therefore, the patients in the low-ICD risk score group are suitable candidates for combination of chemotherapy and immunotherapy. In contrast, the high-ICD-related risk score group bearing a low immune score may not be able to respond to immunotherapy as a treatment. Both *CCNE1* and *PLK1* were highly expressed in the high-risk group compared with the low-risk group, which indicated that targeting therapy based on these two genes may activate the immune system, increase the immune infiltration level, and establish an immune microenvironment for an improved immunotherapy response. Ongoing clinical trials have confirmed that tumor vaccines combined with immune checkpoint inhibitors can improve the immune response in patients with melanoma (Sahin et al., 2020), which is similar to an assumption made in our breast cancer study.

## 5 Conclusion

*CCNE1* and *PLK1* were identified as potential antigens in breast cancer. The ICD-related prognostic model could distinguish the immune heterogeneity of patients with breast cancer and predict prognosis. Patients with high-ICD-related risk scores were suitable to receive combination treatments based on *CCNE1* or *PLK1* and immune checkpoint inhibitors. In the future, these findings will help us develop more accurate treatment schemes for patients with breast cancer.

## Data Availability

The datasets analyzed during the current study are available in The Cancer Genome Atlas database (TCGA, https://portal.gdc.cancer.gov/).

## Footnotes

1 TCGA: https://portal.gdc.cancer.gov.

2 GEPIA2: http://gepia2.cancer-pku.cn.

3 cBioPortal: http://www.cbioportal.org/.

4 TIMER: https://cistrome.shinyapps.io/timer/.

5 TIDE: http://tide.dfci.harvard.edu/.

## References

[B1] Baracco E. E. Petrazzuolo A. Kroemer G. (2019). Assessment of annexin A1 release during immunogenic cell death. Methods Enzymol. 629, 71–79. 10.1016/bs.mie.2019.06.010 31727257

[B2] Bøvelstad H. M. Nygård S. Størvold H. L. Aldrin M. Borgan Ø. Frigessi A. (2007). Predicting survival from microarray data--a comparative study. Bioinformatics 23 (16), 2080–2087. 10.1093/bioinformatics/btm305 17553857

[B3] Cafri G. Gartner J. J. Zaks T. Hopson K. Levin N. Paria B. C. (2020). mRNA vaccine-induced neoantigen-specific T cell immunity in patients with gastrointestinal cancer. J. Clin. Invest. 130 (11), 5976–5988. 10.1172/JCI134915 33016924 PMC7598064

[B4] Cerami E. Gao J. Dogrusoz U. Gross B. E. Sumer S. O. Aksoy B. A. (2012). The cBio cancer genomics portal: an open platform for exploring multidimensional cancer genomics data. Cancer Discov. 2 (5), 401–404. 10.1158/2159-8290.CD-12-0095 22588877 PMC3956037

[B5] Cortes J. Cescon D. W. Rugo H. S. Nowecki Z. Im S. A. Yusof M. M. (2020). Pembrolizumab plus chemotherapy versus placebo plus chemotherapy for previously untreated locally recurrent inoperable or metastatic triple-negative breast cancer (KEYNOTE-355): a randomised, placebo-controlled, double-blind, phase 3 clinical trial. Lancet 396 (10265), 1817–1828. 10.1016/S0140-6736(20)32531-9 33278935

[B6] Di Virgilio F. Sarti A. C. Falzoni S. De Marchi E. Adinolfi E. (2018). Extracellular ATP and P2 purinergic signalling in the tumour microenvironment. Nat. Rev. Cancer 18, 601–618. 10.1038/s41568-018-0037-0 30006588

[B7] Fucikova J. Kralikova P. Fialova A. Brtnicky T. Rob L. Bartunkova J. (2011). Human tumor cells killed by anthracyclines induce a tumor-specific immune response. Cancer Res. 71 (14), 4821–4833. 10.1158/0008-5472.CAN-11-0950 21602432

[B8] Fucikova J. Kepp O. Kasikova L. Petroni G. Yamazaki T. Liu P. (2020). Detection of immunogenic cell death and its relevance for cancer therapy. Cell Death Dis. 11 (11), 1013. 10.1038/s41419-020-03221-2 33243969 PMC7691519

[B9] Fucikova J. Spisek R. Kroemer G. Galluzzi L. (2021). Calreticulin and cancer. Cell Res. 31 (1), 5–16. 10.1038/s41422-020-0383-9 32733014 PMC7853084

[B10] Galluzzi L. Buqué A. Kepp O. Zitvogel L. Kroemer G. (2017). Immunogenic cell death in cancer and infectious disease. Nat. Rev. Immunol. 17 (2), 97–111. 10.1038/nri.2016.107 27748397

[B11] Galluzzi L. Vitale I. Warren S. Adjemian S. Agostinis P. Martinez A. B. (2020). Consensus guidelines for the definition, detection and interpretation of immunogenic cell death. J. Immunother. Cancer 8 (1), e000337. 10.1136/jitc-2019-000337 32209603 PMC7064135

[B12] Garg A. D. De Ruysscher D. Agostinis P. (2015). Immunological metagene signatures derived from immunogenic cancer cell death associate with improved survival of patients with lung, breast or ovarian malignancies: A large-scale meta-analysis. Oncoimmunology 5 (2), e1069938. 10.1080/2162402X.2015.1069938 27057433 PMC4801472

[B13] Golsteyn R. M. Lane H. A. Mundt K. E. Arnaud L. Nigg E. A. (1996). The family of polo-like kinases. Prog. Cell Cycle Res. 2, 107–114. 10.1007/978-1-4615-5873-6_11 9552388

[B14] Greaves M. (2015). Evolutionary determinants of cancer. Cancer Discov. 5 (8), 806–820. 10.1158/2159-8290.CD-15-0439 26193902 PMC4539576

[B15] Grunwitz C. Kranz L. M. (2017). mRNA cancer vaccines-messages that prevail. Curr. Top. Microbiol. Immunol. 405, 145–164. 10.1007/82_2017_509 28401358

[B16] King S. I. Purdie C. A. Bray S. E. Quinlan P. R. Jordan L. B. Thompson A. M. (2012). Immunohistochemical detection of Polo-like kinase-1 (PLK1) in primary breast cancer is associated with TP53 mutation and poor clinical outcom. Breast Cancer Res. 14 (2), R40. 10.1186/bcr3136 22405092 PMC3446374

[B17] Li T. Fan J. Wang B. Traugh N. Chen Q. Liu J. S. (2017). TIMER: A web server for comprehensive analysis of tumor-infiltrating immune cells. Cancer Res. 77 (21), e108–e110. 10.1158/0008-5472.CAN-17-0307 29092952 PMC6042652

[B18] Liu W. W. Li W. D. Zhang Y. J. Zhang M. L. (2021). Regulatory effect of miR497-5p-CCNE1 Axis in triple-negative breast cancer cells and its predictive value for early diagnosis. Cancer Manag. Res. 13, 439–447. 10.2147/CMAR.S284277 33500658 PMC7823138

[B19] Luhtala S. Staff S. Tanner M. Isola J. (2016). Cyclin E amplification, over-expression, and relapse-free survival in HER-2-positive primary breast cancer. Tumour Biol. 37 (7), 9813–9823. 10.1007/s13277-016-4870-z 26810187

[B20] Malaker S. A. Penny S. A. Steadman L. G. Myers P. T. Loke J. C. Raghavan M. (2017). Identification of glycopeptides as posttranslationally modified neoantigens in leukemia. Cancer Immunol. Res. 5 (5), 376–384. 10.1158/2326-6066.CIR-16-0280 28314751 PMC5508727

[B21] McGranahan N. Swanton C. (2017). Clonal heterogeneity and tumor evolution: Past, present, and the future. Cell 168 (4), 613–628. 10.1016/j.cell.2017.01.018 28187284

[B22] Montaudon E. Nikitorowicz-Buniak J. Sourd L. Morisset L. El Botty R. Huguet L. (2020). PLK1 inhibition exhibits strong anti-tumoral activity in CCND1-driven breast cancer metastases with acquired palbociclib resistance. Nat. Commun. 11 (1), 4053. 10.1038/s41467-020-17697-1 32792481 PMC7426966

[B23] Pérez-Núñez I. Rozalén C. Palomeque J. Á. Sangrador I. Dalmau M. Comerma L. (2022). LCOR mediates interferon-independent tumor immunogenicity and responsiveness to immune-checkpoint blockade in triple-negative breast cancer. Nat. Cancer 3 (3), 355–370. 10.1038/s43018-022-00339-4 35301507

[B24] Rausch S. Schwentner C. Stenzl A. Bedke J. (2014). mRNA vaccine CV9103 and CV9104 for the treatment of prostate cancer. Hum. Vaccin. Immunother. 10 (11), 3146–3152. 10.4161/hv.29553 25483661 PMC4514038

[B25] Sahin U. Oehm P. Derhovanessian E. Jabulowsky R. A. Vormehr M. Gold M. (2020). An RNA vaccine drives immunity in checkpoint-inhibitor-treated melanoma. Nature 585 (7823), 107–112. 10.1038/s41586-020-2537-9 32728218

[B26] Schmid P. Rugo H. S. Adams S. Schneeweiss A. Barrios C. H. Iwata H. (2020). Atezolizumab plus nab-paclitaxel as first-line treatment for unresectable, locally advanced or metastatic triple-negative breast cancer (IMpassion130): updated efficacy results from a randomised, double-blind, placebo-controlled, phase 3 trial. Lancet. Oncol. 21 (1), 44–59. 10.1016/S1470-2045(19)30689-8 31786121

[B27] Schmid P. Cortes J. Pusztai L. McArthur H. Kümmel S. Bergh J. (2020). Pembrolizumab for early triple-negative breast cancer. N. Engl. J. Med. 382 (9), 810–821. 10.1056/NEJMoa1910549 32101663

[B28] Schmid P. Cortes J. Dent R. Pusztai L. McArthur H. Kümmel S. (2022). Event-free survival with pembrolizumab in early triple-negative breast cancer. N. Engl. J. Med. Overseas. Ed. 386 (6), 556–567. 10.1056/nejmoa2112651 35139274

[B29] Schumacher T. N. Scheper W. Kvistborg P. (2019). Cancer neoantigens. Annu. Rev. Immunol. 37, 173–200. 10.1146/annurev-immunol-042617-053402 30550719

[B30] Sen S. Won M. Levine M. S. Noh Y. Sedgwick A. C. Kim J. S. (2022). Metal-based anticancer agents as immunogenic cell death inducers: the past, present, and future. Chem. Soc. Rev. 51 (4), 1212–1233. 10.1039/d1cs00417d 35099487 PMC9398513

[B31] Shakeel I. Basheer N. Hasan G. M. Afzal M. Hassan M. I. (2021). Polo-like kinase 1 as an emerging drug target: structure, function and therapeutic implications. J. Drug Target. 29 (2), 168–184. 10.1080/1061186X.2020.1818760 32886539

[B32] Sung H. Ferlay J. Siegel R. L. Laversanne M. Soerjomataram I. Jemal A. (2021). Global cancer statistics 2020: GLOBOCAN estimates of incidence and mortality worldwide for 36 cancers in 185 countries. Ca. Cancer J. Clin. 71 (3), 209–249. 10.3322/caac.21660 33538338

[B33] Tang Z. Li C. Kang B. Gao G. Li C. Zhang Z. (2017). GEPIA: a web server for cancer and normal gene expression profiling and interactive analyses. Nucleic Acids Res. 45 (W1), W98–W102. 10.1093/nar/gkx247 28407145 PMC5570223

[B34] Tesniere A. Schlemmer F. Boige V. Kepp O. Martins I. Ghiringhelli F. (2010). Immunogenic death of colon cancer cells treated with oxaliplatin. Oncogene 29 (4), 482–491. 10.1038/onc.2009.356 19881547

[B35] Turner N. C. Liu Y. Zhu Z. Loi S. Colleoni M. Loibl S. (2019). Cyclin E1 expression and palbociclib efficacy in previously treated hormone receptor-positive metastatic breast cancer. J. Clin. Oncol. 37 (14), 1169–1178. 10.1200/JCO.18.00925 30807234 PMC6506420

[B36] Wang Y. Zhang L. Xu Z. Miao L. Huang L. (2018). mRNA vaccine with antigen-specific checkpoint blockade induces an enhanced immune response against established melanoma. Mol. Ther. 26 (2), 420–434. 10.1016/j.ymthe.2017.11.009 29249397 PMC5835019

[B37] Wang B. Huang X. Liang H. Yang H. Guo Z. Ai M. (2021). PLK1 inhibition sensitizes breast cancer cells to radiation via suppressing autophagy. Int. J. Radiat. Oncol. Biol. Phys. 110 (4), 1234–1247. 10.1016/j.ijrobp.2021.02.025 33621661

[B38] Watkins T. B. K. Lim E. L. Petkovic M. Elizalde S. Birkbak N. J. Wilson G. A. (2020). Pervasive chromosomal instability and karyotype order in tumour evolution. Nature 587 (7832), 126–132. 10.1038/s41586-020-2698-6 32879494 PMC7611706

[B39] Yang R. Xing L. Zheng X. Sun Y. Wang X. Chen J. (2019). The circRNA circAGFG1 acts as a sponge of miR-195-5p to promote triple-negative breast cancer progression through regulating CCNE1 expression. Mol. Cancer 18 (1), 4. 10.1186/s12943-018-0933-7 30621700 PMC6325825

[B40] Yarchoan M. Johnson B. A. Lutz E. R. Laheru D. A. Jaffee E. M. (2017). Targeting neoantigens to augment antitumour immunity. Nat. Rev. Cancer 17 (4), 209–222. 10.1038/nrc.2016.154 28233802 PMC5575801

[B41] Yuan J. Hu Z. Mahal B. A. Zhao S. D. Kensler K. H. Pi J. (2018). Integrated analysis of genetic ancestry and genomic alterations across cancers. Cancer Cell 34 (4), 549–560. 10.1016/j.ccell.2018.08.019 30300578 PMC6348897

[B42] Yuan Q. Zheng L. Liao Y. Wu G. (2021). Overexpression of CCNE1 confers a poorer prognosis in triple-negative breast cancer identified by bioinformatic analysis. World J. Surg. Oncol. 19 (1), 86. 10.1186/s12957-021-02200-x 33757543 PMC7989008

[B43] Zack T. I. Schumacher S. E. Carter S. L. Cherniack A. D. Saksena G. Tabak B. (2013). Pan-cancer patterns of somatic copy number alteration. Nat. Genet. 45 (10), 1134–1140. 10.1038/ng.2760 24071852 PMC3966983

[B44] Zhao Z. M. Yost S. E. Hutchinson K. E. Li S. M. Yuan Y. C. Noorbakhsh J. (2019). CCNE1 amplification is associated with poor prognosis in patients with triple negative breast cancer. BMC Cancer 19 (1), 96. 10.1186/s12885-019-5290-4 30665374 PMC6341717

[B45] Zhu M. Yang M. Zhang J. Yin Y. Fan X. Zhang Y. (2021). Immunogenic cell death induction by ionizing radiation. Front. Immunol. 12, 705361. 10.3389/fimmu.2021.705361 34489957 PMC8417736

