# Supplementary material for: Identification of tumor antigens and immunogenic cell death-related subtypes for the improvement of immunotherapy of breast cancer
Source: Front Cell Dev Biol. 2022 Oct 25;10:962389. doi: 10.3389/fcell.2022.962389 (PMC9641162; doi:10.3389/fcell.2022.962389)
Supplement: Supplementary file 11 [file DataSheet1.docx]

**Supplementary Table 1.** Over-expressed genes, amplified genes with frequency > 0.1% and mutated genes with frequency > 0.1% in breast cancer tissue compared with normal tissue based on GEPIA2 analysis.

**Supplementary Table 2.** Relationship between survival (disease-free survival and overall survival) and 701 gens in breast cancer cohort.

**Supplementary Table 3.** Expression of CCNE1, PLK1 and SERPINA1 in breast cancer tissue and normal tissue.

**Supplementary Table 4.** Connections among immunogenic cell death (ICD)-related genes explored by the protein-protein interaction (PPT) network analysis based on the STRING database.

**Supplementary Table 5.** Expression of the immunogenic cell death (ICD)-related genes between breast cancer tissue and normal tissue.

**Supplementary Table 6.** Correlation between immunogenic cell death (ICD)-related and overall survival analyzed by univariate Cox regression analysis in breast cancer cohort.

**Supplementary Table 7.** The immunogenic cell death (ICD)-related risk score based on the LASSO Cox-regression analysis in breast cancer cohort.

**Supplementary Table 8**. Infiltration of immune cells, expression of 8 immune checkpoints and human leukocyte antigen (HLA) genes in immunogenic cell death (ICD)-related low-and high-risk score groups in breast cancer cohort.

**Supplementary Table 9**. Tumor mutational burden (TMB) score and microsatellite instability (MSI) score, the potential efficacy of immunotherapy based on tumor immune dysfunction and exclusion (TIDE) analysis in immunogenic cell death (ICD)-related low-and high-risk score groups in breast cancer cohort.

**Supplementary Table 10.** Association of immunogenic cell death (ICD) risk signature with Clinical Characteristics in breast cancer cohort.
